# Deciphering the link between PI3K and PAK: An opportunity to target key pathways in pancreatic cancer?

**DOI:** 10.18632/oncotarget.13309

**Published:** 2016-11-11

**Authors:** Kiruthikah Thillai, Hoyin Lam, Debashis Sarker, Claire M. Wells

**Affiliations:** ^1^ Division of Cancer Studies, Kings College London, London, United Kingdom; ^2^ Department of Medical Oncology, Guys and St Thomas NHS Trust, London, United Kingdom

**Keywords:** pancreatic cancer, PAK, PI3K

## Abstract

The development of personalised therapies has ushered in a new and exciting era of cancer treatment for a variety of solid malignancies. Yet pancreatic ductal adenocarcinoma (PDAC) has failed to benefit from this paradigm shift, remaining notoriously refractory to targeted therapies. Chemotherapy is the cornerstone of management but can offer only modest survival benefits of a few months with 5-year survival rates rarely exceeding 3%. Despite these disappointing statistics, significant strides have been made towards understanding the complex biology of pancreatic cancer, with deep genomic sequencing identifying novel genetic aberrations and key signalling pathways. The PI3K-PDK1-AKT pathway has received great attention due to its prominence in carcinogenesis. However, efforts to target several components of this network have resulted in only a handful of drugs demonstrating any survival benefit in solid tumors; despite promising pre-clinical results. p-21 activated kinase 4 (PAK4) is a gene that is recurrently amplified or overexpressed in PDAC and both PAK4 and related family member PAK1, have been linked to aberrant RAS activity, a common feature in pancreatic cancer. As regulators of PI3K, PAKs have been highlighted as a potential prognostic marker and therapeutic target. In this review, we discuss the biology of pancreatic cancer and the close interaction between PAKs and the PI3K pathway. We also suggest proposals for future research that may see the development of effective targeted therapies that could finally improve outcomes for this disease.

## INTRODUCTION TO PANCREATIC DUCTAL ADENOCARCINOMA (PDAC)

Pancreatic ductal adenocarcinoma (PDAC) is a devastating and lethal disease. Epidemiological trends predict that over the next two decades PDAC will overtake breast, colorectal and prostate cancer to become the second leading cause of cancer related mortality worldwide [1]. Due to the surreptitious nature of PDAC formation, the majority of patients present with advanced disease that is inoperable and therefore incurable. Even those few that undergo surgery are likely to succumb to metastatic spread within two years. Despite significant international research efforts, the last twenty years have witnessed the development of only two new chemotherapy regimens, both of which fail to improve survival by more than a few months for advanced disease compared with the previous standard of care, the nucleoside analogue gemcitabine [2, 3]. To date, there are no effective targeted therapies for PDAC, highlighting the need for a better understanding of pancreatic tumor biology and the identification of new exploitable pathways.

By far the most common malignancy to arise from the pancreas, PDAC is a heterogeneous genetic disease with over 60 mutations identified per tumor [4]. Despite this, PDACs have several distinctive features; characteristic mutations in established onco- and tumor suppressor genes, dense surrounding stromal tissue and a high propensity to metastasize [5–7]. PDACs typically arise from one of three histological precursor lesions; pancreatic intra-epithelial neoplasia (PanIN), intra ductal papillary mucinous neoplasm (IPMN) and mucinous cystic neoplasia [8]. Echoing the behavior of other adenocarcinomas, pancreatic cancer develops in a stepwise fashion from low- to high-grade dysplasia before culminating in malignant transformation [9, 10]. This process is accompanied by an increasing frequency of genetic aberrations [11]. The commonest mutation is at codon 12 in the proto-oncogene Kristen rat sarcoma viral oncogene homolog (*KRAS*) [12]. Responsible for encoding a small (~21kDa) GTPase, *KRAS* mutations occur in nearly 100% of PDAC tumors [12]. Often the first gene to be mutated in PDAC, it has also been recurrently identified in pre-invasive lesions and is therefore thought to be involved in carcinogenesis [13–16]. Constitutive activation of KRAS results in sustained and unregulated proliferation, evasion of apoptosis, re-modelling of the micro-tumor environment, increased cell migration and metastatic spread [17–20]. Murine pancreata with *KRAS*^G12D^ or *KRAS*^G12V^ mutations invariably develop dysplastic changes and subsequent invasive metastatic PDAC [16]. Three further genetic aberrations commonly occur in PDAC, namely inactivation of *p16INK4A/CDKN2A* (p16), *Tp53* and *SMAD* family member 4 (*SMAD4*) with a reported prevalence of 50-70%, 55% and 10% respectively in invasive PDAC [15, 21]. Whilst nearly 90% of precursor PanIN lesions harbor a *KRAS* mutation, these three ‘loss of function’ aberrations are less prevalent in pre-cancerous pathology compared with cancer samples, suggesting they occur later in oncogenesis.

Extensive exome sequencing and copy number analyses of 142 early-stage PDAC samples have led to a clearer portrayal of the genomic landscape [14]. 16 genes were found to be recurrently mutated. In addition to established mutations, several genes responsible for modifying chromatic and DNA damage repair were also mutated. Further aberrations in the SLIT/ROBO signaling pathway were identified, implicating axon guidance genes in pancreatic cancer development. A more recent genomic analysis of 456 early stage PDAC samples identified 32 genes that were recurrently mutated, each of which could be assigned to 10 pathways; KRAS, TGF- β, WNT, NOTCH, ROBO/SLIT signaling, G1/S transition, SWI-SNF, chromatin modification, DNA repair and RNA processing [22]. Expression analysis led to the classification of 4 subtypes; squamous, pancreatic progenitor, immunogenic and aberrantly differentiated endocrine and exocrine (ADEX) with clear correlation of histological features.

Whilst it is now clear that PDAC is a complex genetic disease developing from a cascade of mutations in pancreatic cells, this knowledge has yet to have a meaningful clinical impact on PDAC survival. Nevertheless, the identification of key pathways is vital in guiding the search for novel therapeutic targets as well as potential prognostic and predictive biomarkers. A pilot study of 92 patients assessed the feasibility of attaining PDAC tissue for genomic analysis in ‘real-time’ in order to perform genetic screening within a clinically satisfactory time-frame [23]. Three molecular targets were screened for: HER2 amplification, *KRAS* wild type and mutations in DNA damage repair pathway (*BRCA1, BRCA2, PALB2* and *ATM*). 22 patients with these genetic signatures were identified. The study highlighted the potential for personalized therapy using high-quality actionable genomic data but demonstrated the challenges of efficiently screening bio specimens in these patients as well as the need for improved clinical trial options.

## THE PHOSPHATIDYLINOSITIDE 3- KINASE (PI3K) FAMILY

Oncogenic KRAS activates a plethora of effector downstream pathways. The two most significant canonical signaling pathways are mitogen activated protein kinase (MAPK) (see review by Samatar et al for a more detailed description of the MAPK pathway) and the phosphatidylinositide 3-kinase (PI3K) pathway [24]. The PI3K-PDK1-AKT pathway is an intricate signaling network, regulating cell metabolism, growth, migration, survival and angiogenesis and therefore when aberrantly activated, results in oncogenesis [25]. The PI3K family is a group of lipid kinases that phosphorylates the 3′OH group of phosphatidylinositols [26]. There are three classes, each distinguished by structural and functional differences. Class 1 PI3Ks are the best characterized in cancer and primarily responsible for the production of D-3 phosphoinositides in response to various growth factors, and it is against this class that drug development efforts to target this pathway have focused on [27, 28].

### PI3K signaling

**Figure 1:**
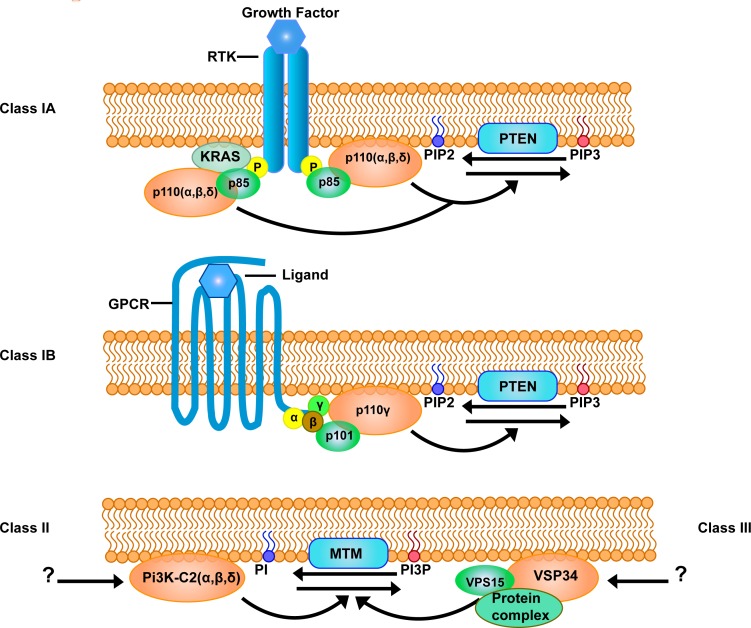
Different classes of the PI3K family The phosphatidylinositol 3-kinase (PI3K) family comprises of three classes with several isoforms which catalyses different substrates. Class I PI3K consists of Class IA and Class IB Pi3Ks, both converting phosphatidylinositide 4,5- bisphosphate (PIP2) into phosphatidylinositide 3,4,5- triphosphate (PIP3). Class IA PI3Ks are activated by receptor tyrosine kinases (RTK) which can activate catalytic isoforms p110α, β and δ through the adaptor subunit p85. There are five known adaptor subunit isoforms, namely p85α, p85β, p55α, p55γ and p50α. Class IB PI3K are activated by G-protein coupled receptors (GPCRs) and has one catalytic subunit p110γ and two known regulatory subunits p101 and p87. Phosphatase and tensin homolog (PTEN) phosphatase inactivates Class I PI3K signaling. Class II and class III PI3Ks are known to catalyze phosphatidylinositol (PI) into phosphatidylinositol 3-phosphate (PI3P). Myotubularin (MTM) phosphatase inactivates both class II and class III PI3K signaling. Class II PI3Ks consists of a catalytic subunit with three isoforms PI3K-C2 α, β, and γ, but does not associate with any regulatory subunits. Class III PI3K is composed of a VPS32 catalytic subunit and a VPS15 regulatory subunit, often bound in a multiprotein complex.

**Figure 2:**
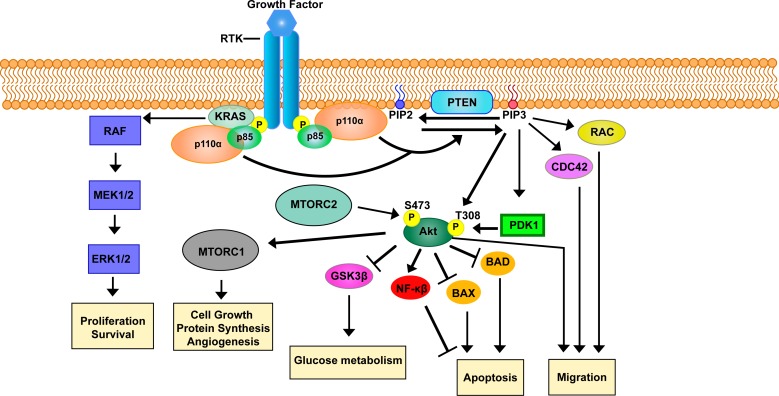
The class I PI3K signaling pathway through receptor tyrosine kinases Upon binding of growth factors to receptor tyrosine kinases (RTKs), the receptor gets activated through the phosphorylation of YXXM motifs. The activation of the RTKs will result into the recruitment of p85 and the p110 subunits, which together forms the phosphatidylinositol 3-kinase (PI3K), to the plasma membrane in order to phosphorylate the phosphatidylinositide 4,5- bisphosphate (PIP2) and transforms it into the phosphatidylinositide 3,4,5- triphosphate (PIP3). Alternatively, the PI3Ks can also be activated by KRAS, which alone can activate downstream signaling pathways such as the RAF-MEK-ERK pathway and result into cell proliferation and survival. The PI3K signaling pathway is normally inactivated through Phosphatase and tensin homolog (PTEN) converting PIP3 back into PIP2. PIP3 acts as a second messenger that can recruit AKT to the plasma membrane and activate downstream effectors such as Rac1, CDC42 and phosphoinositide-dependent kinase PDK1. Activated PDK1 is able to phosphorylate recruited AKT on the Thr308 residue. In addition, MTOR complex 2 (MTORC2) is able to phosphorylate AKT on the Ser473 residue. Phosphorylation of either or both residues of AKT results in downstream signaling events such as cell growth, protein synthesis and angiogenesis through MTORC1. Furthermore, activated AKT can also promote cell survival through inhibiting cellular processes such as glucose metabolism and apoptosis through blocking glycogen synthase kinase 3β (GSK3β) and pro-apoptotic members of the bcl-2 family; BAD and BAX respectively. Moreover, AKT can also inhibit apoptosis through the activation of Nuclear factor κβ (NF-κβ), and promote migration.

Each class 1A PI3K protein contains both a regulatory- (p85) and catalytic-(p110) subunit. p85 has 3 isoforms p85α (p85α, p55α and p50α), p86α and p55γ each encoded by three genes, *PIK3R1, PIK3R2* and *PIK3R3* respectively [29]. The catalytic subunit also has 3 isoforms (p110α, p110β and p110δ) encoded by *PIK3CA, PIK3CB* and *PIK3CD* respectively (Figure 1). It is the regulatory subunit that maintains the catalytic subunit in its less active state in quiescent cells and interacts with phosphotyrosine residues of adaptor proteins or activated growth factor receptors [27]. Somatic mutations of the aforementioned genes can lead to constitutive activation of the PI3K pathway and subsequent malignant transformation of cells [30]. In the absence of mutation, amplification of *PIK3CA* has also been identified as a driver in a number of epithelial tumors [31, 32]. Once activated, PI3K converts the lipid phospatidylinsotide-4,5-bisphophate (PIP_2_) to phosphatidylinositide 3,4,5- triphosphate PIP_3_ (Figure 2). Proteins with pleckstring homology (PH) domains travel to the sites of PI3K activation and bind to PIP_3_. Two specific serine/threonine kinases PDK1 and AKT are subsequently both brought into close proximity of each other by PIP_3_[27]. PDK1 activates AKT by phosphorylation of the threonine site (T308) in AKT. The serine site (S473) can be activated by several kinases including mTOR complex 2 (mTORC2), PDK-1, integrin-linked kinase (ILK) and AKT itself [33–35].

This critical signal between PDK1 and AKT promotes cell growth and survival by various mechanisms including the inhibition of the pro-apoptotic factors BAD and BAX [36]. One of the major downstream effectors of AKT is mTOR complex 1 (mTORC1) which is responsible for growth factor signaling, AMP levels, oxygen availability and nutrition [37]. The tumor suppressor gene phosphatase and tensin homolog deleted on chromosome 10 (PTEN) dephosphorylates PIP3, converting it back to PIP_2_, subsequently terminating this process and is therefore a critical negative regulator of the PI3K pathway. Arguably the most important tumor suppressor gene second only to p53 in many malignancies, loss of PTEN activity can result from mutation, promoter methylation, phosphorylation or delocalization from the plasma membrane [38].

Although there is clear evidence implicating Class1A PI3Ks in PDAC oncogenesis, Class 1B has also been implicated in tumor formation. Indeed, P110ϒ is expressed in patients with pancreatitis suggesting it has a role in inflammation, which in turn is thought to be a precursor for malignancy. Pre-clinical kinetic assays have determined that p110β and ϒ are required for AKT activation [39].

### PI3K pathway in PDAC

The PI3K pathway appears to be critical in the development and maintenance of PDAC. In a recent study of transgenic mice with *KRAS*^G12D^ mutations, PDK1 inactivation prevented PanIN and PDAC formation [40]. The mice had a normal survival with pancreata of normal morphology but signs of cellular stress as expected in the presence of mutated *KRAS*. This finding suggests that PDK1 activity, a pivotal component of the PI3K pathway, is a critical driver of pancreatic dysplasia. This is contrary to a previous report in non-small cell lung cancer, where the RAF proto-oncogene serine/threonine protein kinase was reported to be essential for *KRAS* driven adenocarcinoma of the lung rather than PDK1, the absence of which did not reduce tumor formation [41, 42]. Yet when CRAF was ablated in *KRAS*^G12^ mutated mice, it appeared to have no inhibition on PDAC formation. This suggests that although mutated *KRAS* is a critical oncogene in several malignancies, its effectors vary depending on tissue type, demanding careful evaluation of each downstream pathway in differing cancers [40].

Although whole genome and exome sequencing have confirmed that *PTEN* mutations are uncommon in PDAC samples, *PTEN* inactivation appears to have a significant role. In 54 human PDAC samples, 70% displayed no or low PTEN expression and 68.5% of tumors exhibited at least moderate phosphorylated pan-AKT-Ser^473^ expression compared to surrounding stroma [43]. There was also a significant negative correlation between PTEN expression and pan-AKT phosphorylation suggesting that activation of the PI3K pathway via PTEN loss or AKT activation is an important driver in PDAC. Furthermore, high-resolution array Comparative genomic hybridization (CGH) analysis of 61 primary xenografts or epithelial enriched primary tumor cells revealed a deletion of either one or two copies of the *PTEN* locus in 9/61 samples and gain/amplification of the *AKT2* isoform in 12/61 samples. One samples harbored both genetic events. Transgenic mice with heterozygoys loss of *PTEN* developed highly invasive pancreatic cancers compared with *PTEN* wild type mice. All 11 mice with total loss of *PTEN*, developed rapidly progressive acinar-ductal metaplasia [ADM] and PanIN formation but there were only occasional invasive cancers. However, the lack of invasion is complicated by the early death of these mice which did not survive beyond 3 weeks; probably due to pancreatic insufficiency. Further analysis of the littermates of *KRAS* mutated and *PTEN* heterozygous mice at 4,6,8 and 10 weeks revealed that the pancreas of *PTEN* heterozugous mice exhibited an increased frequency and size of ADM and PanIN lesions, a more profound stromal reaction and moderately elevated phosphorylated AKT. These findings suggest that in *KRAS* mutated PDAC; PTEN can repress PI3K signaling and restrain cancer progression. A further study looking at the role of the PI3K/AKT pathway in PDAC formation, examined mice with a latent oncogenic *PIK3CA* mutation (encoding p110α^H1047R^) allele silenced by a lox-stop-lox (LSL) cassette as a knock-in [40]. Transgenic expression of this mutation resulted in increased PIP_3_ levels in murine pancreata, similar to the expression seen in *KRAS* mutated mice. Histological review of the *PIK3CA* mutated mice revealed a greater presence of ADM and all mice subsequently developed PanIN at a rate similar to *KRAS*^G12D^ mice. Analysis of tissue samples revealed similar expression of several downstream components of the PI3K pathway including phosphorylated AKT-Ser^473^ and AKT-Thr^308^, pan-AKT and phosphorylated GSK3β-S in both models. Aged mice with either a *PIK3CA* or *KRAS* mutation developed PDAC within 800 days with comparable survival times and patterns of metastatic spread. Importantly in the tissue taken from the *PIK3CA* mutated mice, there was no evidence of RAS activation, excluding the possibility of upstream RAS being responsible for driving progression. PDK1 was then inactivated in murine pancreata using floxed PDK1 mice to test the importance of PDK1 in PDAC formation. Total PDK1 inactivation appeared to block PanIN and PDAC development in the *KRAS*^G12D^ mouse models, resulting in normal life expectancy and PI3K/AKT inactivation. Deletion of one PDK1 allele did not alter the prevalence of PanIN and PDAC. Loss of PDK1 also reduced the phosphorylation of AKT at the threonine site and its downstream effector GSK3β-S9.

Given this wealth of evidence supporting a role for the PI3K pathway in disease progression it is perhaps not surprising that PI3K inhibitors have been tested in some of the PDAC model systems. A selective oral pan-class I PI3K inhibitor, GDC0941 has been used to treat mice harboring a *KRAS*^G12D^ mutation [40]. After 14 days of treatment, GDC0941 reduced both PDAC tumor growth and phosphorylation of AKT-Thr^308^. This led to disease stability in contrast with vehicle-treated mice where rapid disease progression was observed. Post-mortem tissue analysis also revealed decreased proliferation markers in the GDC0941 treated tumors. In addition to the *KRAS* mutated mice, primary patient-derived PDAC cells were transplanted orthoptically in to the pancreas of immuno-deficient mice. These mice were subsequently treated with GDC0941 and similar tumor suppressive events were recorded. These studies confirm that the PI3K-PDK1-AKT pathway is an important driver for PDAC.

A key hallmark of PDAC is the dense stroma surrounding the tumor. Cancer cells are encased in a mesh composed of stromal fibroblasts, endothelial cells and immune cells; the roles of which have yet to be fully established [44]. Significant debate surrounds the effect of the stroma on cancer progression. Previously thought to be protective of the tumor and a barrier to adequate drug delivery, recent studies have shown that depleted stroma in transgenic mice led to poorer survival with more aggressive cancers suggesting the stroma may in fact hinder tumor progression [45]. A recent study investigating heterocellular signaling (between the cancer cells and stromal cells) has revealed that *KRAS*^G12D^ PDAC cells secrete growth factors including MFCSF, GCSF cytokines and the growth morphogen sonic hedgehog (SHH) which act on the stromal cell compartment, particularly pancreatic stellate cells (PSC) [46]. Interestingly this study reported that *KRAS*^G12D^ expression induces canonical ERK1/2 activation, but found no evidence that AKT was activated in a cell-autonomous way. However, whilst AKT activation did not appear to be via expression of *KRAS*^G12D^, tumor cell AKT substrates, such as GSK3β, were elevated in the PDAC cells suggesting that *KRAS*^G12D^ activates AKT reciprocally via stimulation of PSC rather than cell-autonomously. This emphasizes the need for future genomic and proteomic studies encompassing heterocellular rather than homocellular signaling in PDAC; where the stroma is present proposing AKT as a potential focal point where reciprocal signaling could occur.

## P-21 ACTIVATED KINASES

Targeting either RAS or PI3K pathways has potential limitations, as there is clear evidence of complex cross-talk between the two [47]. Promising treatment strategies using MEK inhibitors for example, unwittingly activated the PI3K pathway by a negative feedback loop [48]. Whilst novel drugs targeting PI3K and its downstream targets remain in development, several have failed to show benefit in clinical trials. Unless clear oncogenic addiction to therapeutic targets has been demonstrated, the lack of patient stratification using clear predictive biomarkers in these trials may explain these recent failures. It is therefore prudent to consider alternative strategies such as targeting components that can directly and indirectly interact with the PI3K pathway including the PAKs.

**Figure 3:**
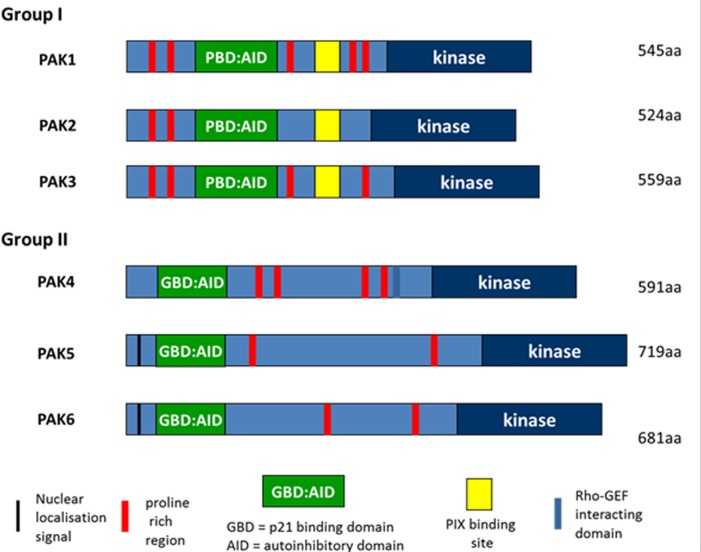
Domain structure of PAK family proteins There are 6 PAK family kinases which are divided into 2 groups. Group I and II PAKS share a common domain consisting of an N-terminal p21-GTPase binding domain (GBD) and a Cterminal serine/threonine kinase domain. Group I PAKS have an Auto inhibitory Domain (AID) overlapping the GBD (GTPase Binding Domain), while the Group II PAKS contain a putative AID sequence. Group I PAKS also contain a PIX binding site in the central region. PAK4 contains a Rho-GEF interacting domain.

There is emerging evidence to suggest that aberrant activation of PAKs can lead to tumorigenesis [16, 49]. Indeed, PAK overexpression has been identified in various solid tumors including breast, colon, prostate and importantly in PDAC [49]. There also appears to be links between PAK activity and the PI3K pathway. The PAK family comprises six non-receptor serine/threonine kinase members, divided into group 1 (PAK1-3) and group 2 (PAK4-6) (Figure 3). Each sub-group has shared and distinguishing features including differences in regulation. Even amongst the subgroups, several distinctions occur. Transgenic knock out (KO) models of PAK5 or PAK6 results in viable healthy litter, whereas PAK4 KO is embryonically lethal [50, 51]. (See reviews by King et al and Radu et al for more information [49, 52]). PAKs are effectors of Rho GTPases Cdc42 and Rac, and upon activation by upstream signaling, mediate several physiological processes[53]. These include cell survival, growth and cytoskeletal dynamics affecting cell adhesion, motility and morphology [54]

### PAK signaling

Phosphorylation of PAK members by PI3K, AKT and PDK1 can modulate various downstream signal events including several cellular process that, when dysregulated could contribute to the hallmarks of cancer [55–57]. Activation of members of the PAK family has been shown to promote cell survival and anti-apoptotic signaling. It has been reported that PAKs promote anchorage independent growth on soft agar *in vitro*, suggesting its contributions to oncogenic transformation [58–62]. Furthermore, aberrant PAK expression via direct phosphorylation of downstream BAD, or indirectly through Raf-1, leads to reduced binding of the death protein BAD to BCL-2 and thus prevents the release of pro-apoptotic factors [63–66]. In addition, there is evidence to suggest PAK2 and PAK4 could inhibit apoptosis independently from BAD phosphorylation through phosphorylation of caspase 7 and inhibition of caspase 8 respectively [67, 68]. PAK1 activates the nuclear factor kappa-light-chain-enhancer (NF-κβ) pathway resulting in resistance to apoptosis and promoting cell growth and angiogenesis [69]. Overexpression of PAK5 inhibits caspase-8 activity and subsequent inhibition of camptothecin induced apoptosis [70].

In addition to survival and anti-apoptotic signaling, PAK is also associated with cell cycle progression and cell proliferation [62, 71–73]. This was demonstrated to be via AKT, ERK and Wnt mediated signaling in colon cancer [74, 75]. PAK1 can also activate the ERK pathway via Mitogen-activated Protein Kinase Kinase (MEK) phosphorylation in a kinase independent manner, with subsequent increased Cyclin D1 expression, leading to cell cycle progression [76]. PAK4 regulates cell cycle progression through phosphorylation of SMAD2/3 of the TGF-β pathway, and by mediating the expression of cell cycle regulators p21, Cyclin D1 and CDC25A [77, 78].

As downstream effectors of the Rho GTPases Cdc42 and Rac, PAKs also play a crucial role in cancer cell migration and invasion, which could contribute toward metastases formation. PAKs are involved in cytoskeletal remodeling through increased cellular contractility via phosphorylation of myosin light chain (MLC), and interaction with microtubules through phosphorylation of GEF-H1[79, 80]. Furthermore, PAKs can induce actin filament elongation and reduce actin disassembly by phosphorylation of LIM kinase (LIMK), and actin polymerization through phosphorylation of p41-Arc, inducing Arp2/3 complex formation [80–82]. In addition, PAKs can modulate lamellipodia and filopodia formation, rapid turnover of focal adhesions by paxillin, and membrane ruffling through filamin A to promote increased cell motility [83–86]. Moreover, cell-cell junctions were found to be inhibited by PAK1 induced Snail activation, promoting dissemination of cancer cells by inducing epithelial mesenchymal transition (EMT) [87, 88]. PAK6, one of the least well characterized family members, is required for hepatocyte growth factor (HGF) activated carcinoma cell-cell disassociation. PAK6 can drive cell-cell disassociation via an IQGAP1/E-cadherin complex leading to the phosphorylation of β-catenin and the subsequent disruption of cell-cell adhesions and β-catenin and PAK6 both localize to cell junctions [89]. PAK6 localization to the cell-cell adhesions occurs via the N-terminus and is Cdc42 dependent [90]. In osteosarcoma and breast cancer cell lines, PAK4 inhibition led to defects in the cell polarization and suppressed β-catenin phosphorylation [91]. Further work needs to be performed to translate these concepts to PDAC cells but they highlight the potential that PAKs have in cell-cell disruption and subsequent dissemination.

It has also been reported that the promotion of invasion through extracellular matrix degradation can be mediated by PAK-induced secretion of matrix metalloproteinases MMP-1, MMP-2, MMP-3 and MMP-9 [77, 92, 93]. Conversely, PAK inhibition leads to reduced migratory and less invasive potential of cancer cells [93–99].

Several reports have suggested that PAKs are also involved in other cellular processes such as vasculogenesis, angiogenesis and metabolic activity [100–103]. However, it is important to note that PAKs have also demonstrated numerous non-kinase dependent functions [66, 68, 76].

### PAKs and cancer

Overexpression of PAKs 1,2,4 and 6 appear to correlate with a more aggressive phenotype of cancer suggesting its potential as a prognostic biomarker [104–109]. In a study using 153 paraffin embedded tissue samples retrospectively collected from patients with advanced ovarian cancer, there was a significant association between increased PAK4 expression and reduced overall and disease free survival, reduced chemo-sensitivity and an increased presence of metastases [77]. PAK1 on chromosome 11q13 and PAK4 on chromosome 19q13.2 are the most commonly overexpressed members of the PAK family in malignancy. Amplified 11q13 and subsequent PAK1 expression has been identified in ovarian cancer and in breast cancer where 11q13 amplification is associated with poor prognosis. Increased PAK1 expression is also present in *BRAF* wild type melanoma compared with *BRAF* mutated disease. Chromosome 19q13.2 is recurrently amplified in PDAC, oral squamous cell carcinoma, breast cancer and ovarian cancer [52].

Even in the absence of genetic amplification, down-regulation of micro RNA 7 (miR-7) has led to overexpression of PAK1 *in vitro* [108, 110–114]. Similarly, PAK2 and PAK4 expression in cancer cells *in vitro* were reported to be negatively mediated by miR-224 and miR-145 or miR-199a/b3p respectively, suggesting a regulatory role for microRNAs on PAK expression [115–117].

### PAK and PDAC

Whilst the exact role of PAKs in the development of PDAC has yet to be established, higher levels of PAK1 and PAK4 have been reported in PDAC. A study looking at 202 human PDAC tissue samples found that PAK1 was overexpressed in 82% of them [118]. Furthermore, a recent study of 72 paraffin-embedded primary pancreatic cancer samples and 20 liver metastases from PDAC patients, reported that PAK1 expression was elevated in primary pancreatic cancer tissue compared with that taken from metastases [117]. PAK1 expression appeared to correlate with improved overall survival (high PAK1 expression was associated with an increased median overall survival of 23.3 months compared with 12.0 months; p=0.004; hazard ratio for death = 0.4). However, there was no correlation with PAK1 expression and tumor stage or clinical characteristics. There are limitations to interpreting this data as it is a retrospective analysis and further prospective work is needed to validate these findings. Interestingly, these studies do not reflect *in vitro* reports where high levels of PAK1 expression are consistently associated with a more aggressive phenotype. Indeed, over expression of PAK1 in the PDAC cell line MiaPaCa2 resulted in increased proliferation, larger number of colony formation and increased migration [119]. Whilst in KRAS wild-type pancreatic cancer cell lines PAK1 overexpression led to increased colony formation. Furthermore, PAK1 depletion led to reduced migration and invasion suggesting PAK1 may play a more prominent role in KRAS-independent PDAC. In addition, a further study also confirmed that PAK1 depletion led to reduced proliferation in the PDAC cell lines MiaPaCa2 and Panc1 [120]. Thus, whilst it is clear that PAK1 over expression is present in PDAC tissue compared with normal tissue, its precise *in vivo* role in carcinogenesis and metastatic spread needs to be investigated further.

PAK4, a member of the group 2 PAKs, is also overexpressed in PDAC. CGH analysis on cDNA microarray of 13 PDAC cell lines identified 24 independent amplicons. Copy number increases were frequently seen at three regions of chromosome 19 (19p13.3, 19q13.1 and 19q13.3). Analysis of these loci revealed that PAK4 at 19q13.1 was frequently expressed at higher levels, correlating with increased copy number changes [113]. A subsequent study using representational oligonucleotide microarray studies analyzed 92 PDAC samples [121]. 22 PDAC cell lines, 26 surgically resected early stage PDAC samples and a further 24 xenografts of surgically resected PDAC samples were used to assess copy number variations. To increase sample size, data from the analysis of 16 primary PDAC tissue samples and 4 cell lines that had previously been published were added to the new samples resulting in a total of 92 individual PDAC samples. Several previously established genetic amplifications and deletions were identified including *Tp16/CDKN2A (Ink/Arf), TP53, c-MYC, KRAS2, MADH4, TERT, EGFR, ERBB2* and *znf217* as well as focal amplification at 19q13 in 5 patient samples and 2 cell lines. Candidate oncogenes from this region were then mapped and using immunohistochemical labeling, PAK4 was identified as being at the ‘epicenter’ of this region. In all samples harboring genomic amplification at 19q13, PAK4 protein expression was higher than normal. To determine the relationship between PAK4 and mutated *KRAS*, the *KRAS2* gene was sequenced in the tumor samples with PAK4 amplification. Codon 12 was mutated in 4 of the 5 samples and 3 of these samples also had genomic amplification and overexpression of KRAS2. These results led the authors to suggest that the PAK4 and RAS pathways may be positively associated. However, one sample had no mutation in *KRAS2* or *PAK4*, thus PAK4 amplification may also occur in the absence of KRAS activation.

qRT-PCR has also revealed increased PAK4 expression in multiple PDAC cell lines with or without the amplification suggesting PAK4 expression is not always dependent on genetic overexpression [122]. Further investigation revealed that reduced PAK4 expression in pancreatic cancer cells leads to a significant reduction in anchorage independent growth. Moreover, expression of a constitutively activated PAK4 protein promoted increased migratory capacity which could be attenuated by reduced PAK4 expression [123]. Although current studies demonstrate an oncogenic role of PAKs in PDAC, more research is needed to identify their exact mechanism and contribution towards PDAC progression. Nevertheless, these findings suggest the PAKs have a central role in the maintenance of PDAC, highlighting their potential as therapeutic targets.

### PAK and the PI3K pathway

**Figure 4:**
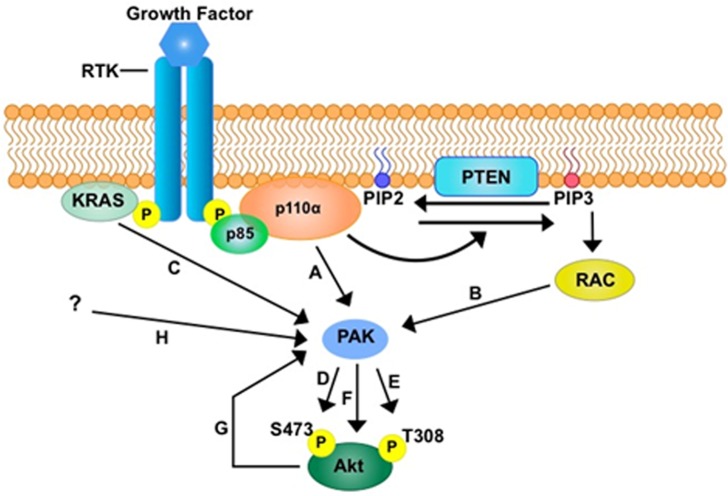
The potential role of PAK in PI3K signalling PAK could be a key mediator in the PI3K-AKT signalling axis through direct activation by PI3K **A**. and through the activation via PIP3-RAC1 **B**. Furthermore, direct activation of PAK through KRAS has been suggested **C**. Activation of PAK has been shown to activate AKT in kinase dependent manners through the phosphorylation of either S473 **D**. or T308 **E**. or both residues, and through a kinase independent manner **F**. The possibility of a negative or positive feedback loop by AKT on PAK **G**. has not been investigated thus far. Other factors that could activate PAK are yet to be identified **H**.

There is evidence that the PAK family can interact with various components of the PI3K pathway (Figure 4). Indeed, qPCR analysis in oral squamous cell carcinomas (OSCC), revealed co-existing PIK3CA and PAK1 amplification in 37% of recurrent tumor samples[124]. PI3K is known to stimulate the small G protein Rac, which in turn is a direct activator of both PAK1 and AKT[56]. In colorectal cancer (CRC) cells, reduced PAK 1 expression led to reduced AKT phosphorylation which correlated with a subsequent decrease in cell proliferation, migration, invasion and survival[75]. A link between PAK and AKT has also been reported in monkey fibroblast like (COS-1) cells where expression of a dominant negative mutant of PAK1, inhibited AKT1 phosphorylation [125]. In addition, the PAK1 kinase domain serves as a scaffold allowing AKT stimulation by PDK1, and aids recruitment of AKT to the cell membrane. Subsequent silencing of PAK1/PAK2 expression also reduced activation of endogenous AKT confirming that PAK1 and PAK2 are required for phosphorylation of AKT. Surprisingly over expression of PAK1 kinase negative mutants also effectively led to the phosphorylation of AKT, suggesting that PAK1 can drive activation of AKT in a kinase independent manner. These studies also demonstrated that AKT1 could be co-immunoprecipitated with PAK1. Indeed, AKT co-immunoprecipitation with PAK1 is also reported in platelet cells further confirming the close interaction between PAK and AKT [126]. In breast cancer cells, endogenous levels of Rac1, PI3K and PAK1 were all stimulated following treatment with epidermal growth factor (EGF) leading to increased migration[127], however in cells expressing a PAK1 kinase dead mutant this migratory response to EGF was significantly diminished. Interestingly, the non-specific PI3K pathway inhibitor LY294002 reduced phosphorylation of PAK1 in these cells placing PAK1 downstream of PI3K suggesting there may in fact be a feedback loop by which the two interact. Recently increased PAK1 expression was associated with activated AKT with a trend towards significance (p=0.05) in human squamous cell skin cancer (SCC) samples [128]. Transgenic mice harboring *KRAS*^G12D^ mutations were bred with PAK1 knock out mice to produce mice that were wild type, heterozygous or knockout for PAK1. The control mice, K5-*rTA*::tet-*KRAS*^G12D^, developed irregular lesions on the skin as early as five days which invariably transformed to SCC malignancies. The Pak1^+/-^ mice took 10 days to develop skin lesions compared to control mice and the Pak^-/-^ mice took 25 days. The PAK^-/-^ mice also had significantly longer survival. In the *KRAS*^G12D^ mice, the presence of PAK1^-/-^ led to a decrease in phosphorylated AKT and downstream targets, mTOR, p70, S6K and S6. Interestingly, two PAK inhibitors, (PF03758309 that suppresses groups 1 and 2 PAK and FRAX-597 that suppresses group 1 PAK) led to marked tumor regression in the K5-*rTA*::tet-*KRAS*^G12D^ mice. Western-blot analysis of tumor samples revealed a reduction in threonine phosphorylated AKT. However, treatment with the AKT inhibitor GSK690693 did not deliver tumor regression thus the importance of AKT signaling in this context remains to be elucidated. There is also evidence that PAK can be mediated by PI3K independent from RAS activity. In breast cancer cells with both *Her2* amplification and *PIK3CA* mutations, treatment with a pan-PI3K inhibitor GDC0941 led to reduced AKT and Rac1/ERK activity [47]. RAS activity was paradoxically not suppressed but induced, suggesting that loss of ERK signaling can occur via a RAS independent pathway. Whilst loss of KRAS via SiRNA knockdown failed to suppress ERK phosphorylation in T47D cells (supporting the theory that this event can occur independently of KRAS activity) in a pancreatic cancer cell line, KRAS knockdown suppressed phospho-ERK activity suggesting that there may be heterogeneity in these pathways depending on tumor type.

In contrast there have been specific studies of PAK4 pathways in PDAC with evidence that PAK4 can modulate the activity of AKT. In pancreatic cancer cell lines stable PAK4 depletion led to a significant decrease in phosphorylated AKT levels [124]. Moreover, PAK4 depleted cells exhibited reduced proliferation and increased levels of cell death with a concomitant decrease in the accumulation of NF-kβ within the nucleus. In MDA-MB-231 breast cancer cells, elevated levels of PAK4 expression correlated with an elevated level of phosphorylated AKT and a more invasive phenotype [129]. Consequently, PAK4 depletion by SiRNA knockdown resulted in reduced phosphorylated AKT and reduced activity of mammalian target of rapamycin (mTOR), a downstream effector of AKT. In contrast a kinase active PAK4 (S474E) enhanced PI3K/AKT signaling. Interestingly a kinase inactive mutant of PAK4 (K350A/K351A) continued to up drive increased levels of phosphorylated AKT suggesting the activity of PAK4 may be both kinase dependent and independent. A study of cisplatin resistant (CDDP) gastric cancer cells also confirmed that in PAK4 SiRNA depleted cells there is a reduction in phosphorylated AKT, whilst total AKT levels were unchanged [130]. PAK4 silenced CDDP gastric cancer cells were then subcutaneously injected into nude mice. Analysis of subsequent tumors with immunohistochemical staining revealed there was reduced expression of phosphorylated AKT in PAK4 depleted compared with wild type tumors. Conversely, treatment with the PI3K inhibitor LY294002 in the wild type CDDP gastric cancer cells, led to a reduction of phosphorylated PAK4 compared with control cells without affecting total PAK4 expression. This was not reproduced with a MEK/ERK inhibitor. These results suggest that PAK4 and the PI3K/AKT pathway can reciprocally activate each other.

## TARGETING THE PI3K AND PAK PATHWAY IN PDAC

Despite a paradigm shift toward molecularly targeted therapies in many solid malignancies, management of advanced PDAC has largely been left unaffected by these changes and response to cytotoxic chemotherapy is poor. Two chemotherapy regimens have improved survival of advanced PDAC in recent years. FOLFIRINOX, a triplet combination which offers a median overall survival (OS) of 11.1 months compared with 6.8 months for gemcitabine alone in a phase III trial (*p*<0.001) [3]. An alternative treatment regimen is Nab-Paclitaxel and gemcitabine which improved median OS to 8.5 months compared with 6.7 months for gemcitabine (*p*<0.001) [2]. The only phase III trial of a targeted therapy to meet its primary endpoint of improved survival was the anti-epidermal growth factor (EGFR) small molecule inhibitor erlotinib, which when combined with gemcitabine offered a marginal survival benefit of a couple of weeks [131]. Unlike lung cancer, the presence of an EGFR mutation was not predictive of response. Yet analysis of patients who experienced cutaneous toxicity revealed that the presence of a skin rash was associated with a higher likelihood of achieving disease control (*P* = .05) after other prognostic factors were controlled for. These results seemed to suggest that there may be a cohort of patients who responded to therapy, highlighting the need for predictive biomarkers. The failure of molecularly targeted therapies so far suggests that patients need to be stratified by genomic expression and treated according to suspected oncogenic drivers. Therefore, the search for more effective therapy has fueled the evaluation of several novel targets for PDAC.

The RAS/PI3K/PDK1/AKT pathway is an exciting target. Targeting RAS has proven challenging with previous attempts to inhibit *KRAS* unsuccessful due to the inability to find an allosteric binding point on mutated RAS. This is likely due to its high binding affinity with GTP. Post-translational modification of RAS proteins (required for membrane anchorage) using farnesyl-transferase inhibitors failed to improve survival in clinical trials when used in conjunction with chemotherapy [132]. Further attempts using an orthosteric inhibitor of the RAS-SOS interaction are promising but have yet to make it to clinical trials [133]. Small molecules that can irreversibly bind to a common mutant *KRAS* ^(G12C)^ are in development. The discovery of a new allosteric regulatory site meant these novel inhibitors bind to a newly discovered regulatory site leading to disruption of both switch-1 and switch-2 leading to reduced GTP binding and subsequent activity [134]. Further studies are warranted to determine clinical activity of these agents. Targeting a protein upstream in prominent oncogenic pathways can be a double-edged sword with normal cellular function at risk of unwarranted inhibition, resulting in potential toxicities. Therefore, research has focused on down-stream targets. Careful selection of the right constituent of the pathway is paramount to maximize efficacy without compromising safety.

### Targeting PI3K pathway

Blockage of the PI3K pathway has yielded some promising results. Several agents that target various components of the PI3K pathway are in development (Table 1). These include pan-Class1 and isoform-specific PI3K inhibitors, dual PI3K -mTOR inhibitors, pan and isoform-specific AKT inhibitors and allosteric mTOR, AKT and PDK1 inhibitors [135]. The PDK1 inhibitor BX912, the dual class 1 PI3K-mTOR inhibitor NPV-BEZ235 and the AKT inhibitor MK-2206 all show evidence of blocking acinar-ductal metaplasia [136] formation in primary PDAC xenografts. NVP-BEZ235, demonstrated inhibited tumor growth in orthoptic pancreatic xenografts [137]. The potent and selective oral pan-class 1 PI3K inhibitor GDC0941 effectively inhibited the growth of primary *KRAS*^G12D^ murine and primary human patient derived PDAC cells *in vivo*. In KRAS mutated mice, GDC0941 efficiently blocked tumor growth with decreased cell proliferation after 14 days of treatment [40]. Treated cells showed a reduction in the phosphorylation of AKT-Thr^308^ highlighting AKT activity as a potential surrogate marker for disease response. GDC0941 has been evaluated in an early phase study, with confirmation of pharmacodynamics response and preliminary evidence of efficacy [138]. Ongoing trials are underway with GDC0941 and other pan-Class I PI3K inhibitors, but are unlikely to yield meaningful responses as single agents due to complexity of feedback loops and crosstalk across linked signaling pathways.

**Table 1:** Table of drug inhibitors targeting the PI3K-PAK-Akt pathway in cancers

Drug	Developed by	Target	Clinical Trials	Ref
GDC0941 Pictilisib	Genentech /Roche	Pan class 1 PI3K inhibitor	Completed Phase I/II trials for breast cancer, non-small cell lung cancer, non-Hodgkin's lymphoma and advanced solid cancers.	[138]
BX912	Berlex Bioscience	PDK1 inhibitor	N/A	[148]
NVP-BEZ235	Novartis	PI3K-mTOR inhibitor	Completed Phase I/II trials for breast cancer, leukemia, castrate resistant prostate cancer, pancreatic neuroendocrine tumors and advanced solid tumors	[149]
MK2206	Merck	Allosteric Pan-AKT inhibitor	Active Phase I/II trials for breast cancer, colon cancer, endometrial carcinoma, pancreatic adenocarcinoma, prostate cancer and renal cell carcinoma. Completed Phase I/II trials for breast cancer, acute and chronic myeloid leukemia, lung carcinoma, ovarian sarcoma, pancreatic acinar carcinoma, pancreatic neuroendocrine tumors and advanced solid tumors.	[150]
AZD6244 Selumetinib	Array BioPharma (Licensed by Astrazeneca)	MEK1/2 inhibitor	Completed/Active Phase I/II trials for breast cancer, lung cancer, melanoma, pancreatic adenocarcinoma and solid tumors	[151]
GSK- 1120212	GlaxoSmithKline	MEK1/2 Inhibitor	Completed/Active Phase I/II/III trials for lung cancer, melanoma, recurrent leukemia and solid tumors	[152]
GSK 690693	GlaxoSmithKline	AKT inhibitor	N/A	[153]
FRAX597	Scripps Research Institute	PAK1 inhibitor	N/A	[154]
PF-3758309	Pfizer	PAK4 inhibitor	N/A	[144]
KPT-7189	Karyopharm	PAK4 inhibitor	N/A	[145]
LY294002	Eli Lilly	Reversible PI3K inhibitor	Active Phase I for neuroblastoma	[155]
NVP-BKM120	Novartis	Pan class I PI3K inhibitor	Active Phase III for metastatic breast cancer	[156]

Dual inhibition to counteract this cross-pathway activation is therefore likely to yield more effective results. The MEK1/2 inhibitor, AZD6244 was tested in combination with two PI3K inhibitors NVP-BKM120 or GDC0941 in transgenic mice and PDAC cell lines. Although MEK inhibition alone was cytostatic, the combination with either PI3K inhibitor led to apoptosis. In *KRAS*^G12D^ mice, dual inhibition delayed PDAC formation and improved survival although responses were not durable [139]. The combined use of the MEK inhibitor GSK1120212 and the AKT inhibitor GSK690693 resulted in statistically significant synergy in PDAC cell lines with decreased phosphorylation of the downstream effector of AKT, RPS6, compared with either agent alone suggesting AKT inhibition could potentiate the response of targeting MAPK [140].

### Targeting PAK

PAK inhibitors have also demonstrated modest efficacy in cell lines although preliminary uses of group 1 PAK inhibitors were largely unsuccessful. Group 1 PAKs have a large ATP binding pocket with high conformational flexibility that likely impeded the identification of high-affinity ligands [141, 142]. However, the PAK1 inhibitor FRAX597 has shown evidence of decreasing PDAC cell proliferation, migration and survival. In addition when combined with the chemotherapeutic agent gemcitabine, FRAX597 synergistically inhibited PDAC proliferation both *in vivo* and *in vitro* [120].

PAK4 is also a promising target. PF-3758309 is a potent (Kd=2.7nM), ATP competitive, pyrolopyrazole inhibitor of PAK4 [143]. As selective downstream targets of PAK4 are unknown, a PAK4 specific assay was constructed with an inducible expression of a PAK4 catalytic domain in cells that are also overexpressing GEF-H1 allowing PAK4 phosphorylation of GEF-H1 to be readily detected; which was shown to be suppressed by PF-3758309 (IC_50_=1.3+/- 0.5nM). Subsequently, PF-3758309 was found to inhibit cellular proliferation and anchorage independent growth across a panel of 92 cell lines including 28 PDAC lines. In the PDAC cell lines, the IC_50_ ranged from 12nM (Panc0813) to 2380 nM (HS700T) although it is not known whether this correlates with PAK4 expression. In tumor xenografts of colon, breast, lung, melanoma and stomach, PF-3758309 impaired tumor growth with a plasma EC_50_ of 0.4nM.

Pre-clinical results were encouraging and PF-3758309 was subsequently tested in a phase 1 study of 33 patients with advanced cancer, including two with PDAC [144]. This was the first and only clinical trial of a PAK4 inhibitor thus far. Patients were treated with the drug orally in a standard 3+3 design and the median number of cycles was 3 (1-10). 9 patients had grade 3 or 4 adverse events and 2 had serious adverse events (abdominal pain and hemoptysis). 1 patient died from progressive disease whilst on treatment. The pharmacokinetic data revealed that after reaching Cmax within 5 hours of dosing, PF-3758309 plasma concentration reduced in a multi-exponential manner with an average terminal half-life of 12.4 to 17.8 hours across doses. There was an absence of dose proportionality in the dose range of 10 to 60 mg, which the investigators suggested was likely due to inter-patient variability. There were no tumor responses seen and the study was terminated due to unwarranted pharmacokinetic findings which were thought to be a result of excessive drug efflux, leading to off-target effects. Yet despite these disappointing preliminary results, the development of more specific PAK inhibitors are underway. It may be that improved specificity can reduce some the off target effects seen with the Pan-PAK inhibitor. In addition, understanding the link between PAK and the PI3K pathway may help identify predictive biomarkers which would help to better identify those patients who may benefit from targeted PAK therapy.

A small molecule PAK4 allosteric modulator KPT-7189 has been shown to suppress PAK4 protein expression in PDAC cell lines, with associated significant reduction in cell proliferation [145]. Moreover, KPT-7189 inhibited the spheroid forming ability of PDAC cells with suppression of EMT and CSC markers. Pre-clinical efficacy studies using a similar anti-PAK4 therapeutic, KPT-7189 with KRAS mutated mice are on-going.

There is also evidence that PAK inhibitors may be used in combination with traditional chemotherapy or used in disease that is chemo-refractory. In 3 PDAC cell lines, Capan-2, PANC1 and SNU-410 that were found to be resistant to gemcitabine, the addition of the PAK4 siRNA appeared to have an effect on cell viability compared with SIRNA silencing of PAK4 or chemotherapy alone suggesting the combination of chemotherapy and PAK4 inhibition warrants further investigation[146]

Ideally, any targeted anti-cancer treatment will be used alongside known predictive biomarkers to identify patients that will benefit from treatment. A disease as complex as PDAC, is likely to require tailored management rather than a ‘one hat fits all’ approach. The failure of several targeted agents in clinical trials is disappointing but perhaps not surprising given the heterogeneous nature of this disease. It is possible that drugs will only work in a sub-group of patients if their complementary pathway is aberrantly activated and pre-determined biomarkers are needed to ensure that potential positive responses are not diluted in large clinical trials. Furthermore even when an effective target is identified, clinical responses are often followed by progression due to the development of resistance as seen in patients with *BRAF* mutated melanoma treated with *BRAF* mutant inhibitors [147]. Therefore, a greater understanding of pertinent pathways and novel targets are warranted.

PAK expression in the tumor tissue and genomic amplification may provide both a prognostic and predictive biomarker for novel therapies. For example, if PAK inhibition can modulate the PI3K pathway, activity of AKT may prove to be surrogate marker of efficacy. Conversely, elevated PAK expression may help identify patients that would benefit from inhibitors of the PI3K pathway.

## FUTURE DIRECTIONS

PDAC is a devastating cancer with a lack of effective therapies for patients with advanced disease. Whilst KRAS mutations occur at a frequency of nearly 100% in PDAC, the challenges of inhibiting RAS have meant efforts have been diverted to unraveling the interacting pathways both downstream from RAS, and those which are independent from RAS. The PAKs have been identified as a potential prognostic marker in several malignancies and their proposed links to the PI3K pathway warrant further attention. Despite the disappointment of several negative clinical trials, great strides have been made in improving our understanding of PDAC biology. This enhanced knowledge will hopefully translate into the development of effective targeted therapies, finally resulting in a much-needed improvement in the treatment of patients with PDAC.
